# Detecting hybridization between sister species of *Terebratulina* (Brachiopoda, Cancellothyridoidea) in the North Atlantic: morphology *versus* molecules

**DOI:** 10.1038/s41598-017-09195-0

**Published:** 2017-08-18

**Authors:** Carsten Lüter, Nina A. Ebeling, Martin Aberhan

**Affiliations:** Museum für Naturkunde, Leibniz Institute for Evolution and Biodiversity Science, Invalidenstraße 43, 10115 Berlin, Germany

## Abstract

Investigating samples of the cancellothyridid brachiopod *Terebratulina* collected during the IceAGE (Me85/3) expedition of RV METEOR at the continental shelf around Iceland with both morphometrical and molecular methods, we were for the first time able to detect a hybridization event between brachiopod sister species, which are thought to have separated 60 MYA. *Terebratulina retusa* and *T. septentrionalis* can clearly be distinguished on the basis of consistent species-specific molecular signatures in both mitochondrial and nuclear markers, whereas morphometrical analyses proved to be less reliable for species determination than previously thought. Two out of 28 specimens were identified as offspring of a one-way hybridization event between *T. retusa* eggs and *T. septentrionalis* sperm. Whereas the fossil record of *Terebratulina* in the North Atlantic region is too fragmentary to reconstruct the history of the hybridization event, the different life history traits of the two species and current oceanographic conditions around Iceland offer plausible explanations for the occurrence of crossbreeds in this common brachiopod genus.

## Introduction

Hybridization has traditionally been regarded as rare in animals but its importance and commonness may have been underrated. With ca. 10% of species being affected^1^, it seems to be a rather widely spread phenomenon in the animal kingdom including marine lophotrochozoans^2–5^. Hybridization is also discussed as a potentially important evolutionary process that drives speciation^6^, but its prevalence remains controversial. In this study, for the first time to our knowledge, we present evidence for hybridization in two brachiopod sister species.

The brachiopod genus *Terebratulina* comprises more than 30 extant species, two of which, *Terebratulina retusa* and *T. septentrionalis*, are common members of invertebrate benthic shelf communities in the temperate to boreal North Atlantic. According to molecular phylogenies *T. retusa* and *T. septentrionalis* in a comprehensive study on molecular systematics of cancellothyridid brachiopods^7^ were found to be sister species resulting from an allopatric speciation event during the Early Paleogene about 60 MYA, which became possible after the opening of the North Atlantic. Whereas *T. retusa* was originally described from Scandinavian waters by Linné^8^, the morphologically similar *T. septentrionalis* was first mentioned in Couthouy’s account on new molluscs from New England’s coast^9^. Ever since Couthouy’s description the sister species were traditionally attributed to either European (*T. retusa*) or North American (*T. septentrionalis*) clades, although several descriptions of *T. septentrionalis* from Northern Scandinavia, Greenland and Iceland existed in the literature^10–15^. Because determining the distribution of either species was severely hampered by their morphological similarity, Curry & Endo^16^ used principal component analyses of shell characters to discriminate between the two. Their more than 2000 specimens clearly fell into two distinct groups mainly distinguishable by the width of their shell ribs. Ribs occur in higher numbers in *T. septentrionalis* and were already used by Davidson^17^ for a separation of Atlantic *Terebratulina* species. Additionally, Curry & Endo^16^ were interested to see whether they would be able to identify intermediate forms, which had been described e.g. from the Danish Gothaab- and Ingolf-Expeditions by Elise Wesenberg-Lund^13–15^ and which she could not attribute to either species, due to a “confusion of characters of both of them, which really made specific identification and description impossible” (ref. 15: p. 9). This potential cline linking the two species could either be interpreted as the morphospace of a single Atlantic species of *Terebratulina* with its two extremes erroneously described as different species (see refs 18–20) or as the product of hybridization^15^. Curry & Endo^16^, unable to find such cline in their data, concluded that hybrids do not exist and that their morphometric approach clearly discriminated between two Atlantic species of *Terebratulina*. This was also confirmed by allozyme data and mitochondrial RFLP analysis^21^ and by sequence analyses of mitochondrial DNA^7^.

The geographical distribution of both species as reconstructed by Curry & Endo^16^ provided another interesting aspect: whereas *T. retusa* was restricted to the East Atlantic and the Mediterranean in their analysis, *T. septentrionalis* seemed to be more widespread from North America to Greenland, Iceland and the Norwegian coast with a possible ice age relict population in Finnmarken (North Norway). Despite partial sympatry no morphological overlap existed in the data of Curry & Endo^16^ between *T. retusa* and *T. septentrionalis* supporting the hypothesis of two valid species of *Terebratulina* inhabiting the North Atlantic. However, the intermediates observed by Wesenberg-Lund^13–15^ remained elusive and her dubious specimens have never been subjected to a rigorous analysis.

In 2011 the IceAGE project collected marine benthos around Iceland with the German research vessel METEOR. Among the samples were 28 specimens of *Terebratulina* suitably preserved for molecular analysis from the geographical region where the unidentifiable specimens of Wesenberg-Lund^15^ had been collected. Sitting half way between mainland Europe and Greenland on top of the mid-Atlantic ridge, Iceland is crucial to understand the biogeographical distribution of *T. septentrionalis* because it represents the most prominent contact zone with its East Atlantic sister taxon *T. retusa*. The IceAGE material enables us to show not only that *T. retusa* in Iceland is much more variable in shell ornamentation than Curry & Endo^16^ suggested, but also that Wesenberg-Lund legitimately struggled to identify her confusing specimens. In particular, we found molecular evidence for hybridization between *T. retusa* and *T. septentrionalis*.

## Results

### Morphology

**Figure 1:**
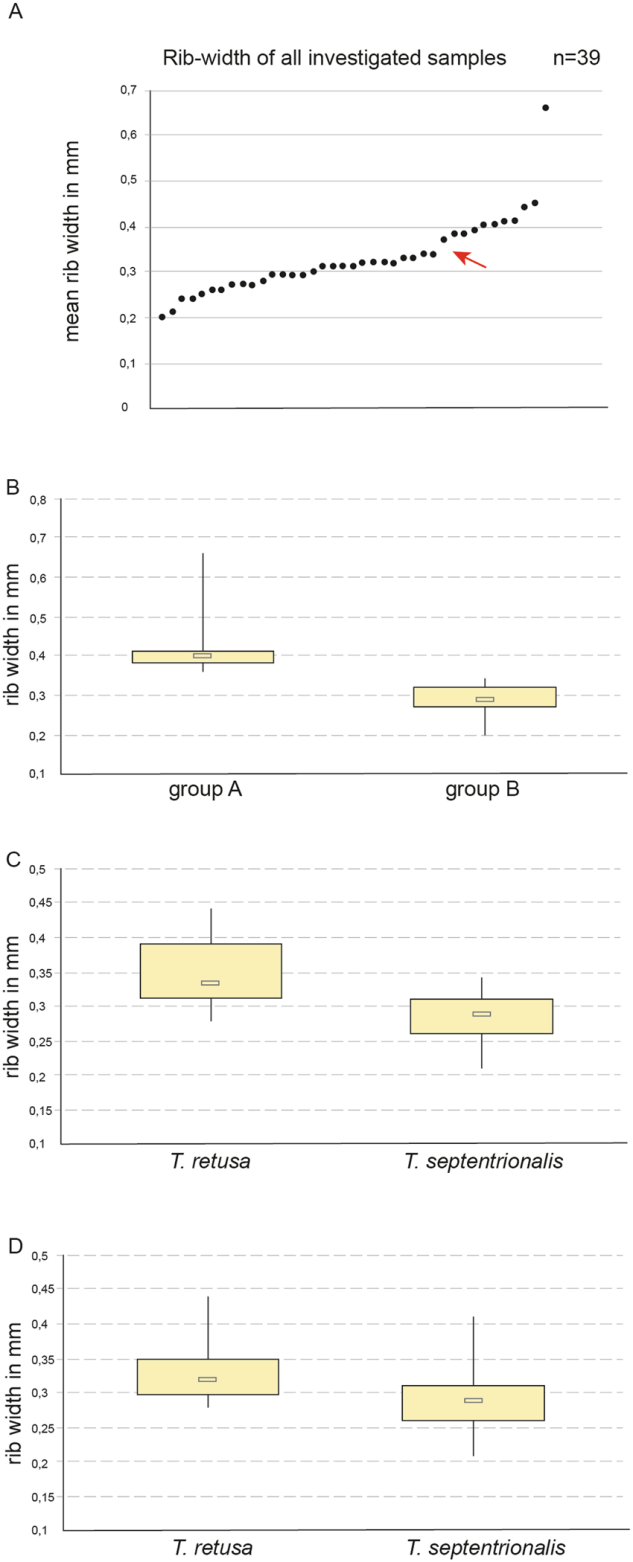
Measurements of shells (rib width) of *Terebratulina* specimens examined in this study. Boxes in (**B**–**D)** depict data between the 25^th^ and the 75^th^ percentiles, vertical lines illustrate the full range of data. (**A**) Mean width of shell ribs of all investigated specimens (n = 39; 23 IceAGE samples supplemented by 16 shells from the brachiopod collection of the Museum für Naturkunde, Berlin). Note the discontinuity between two clusters at about 0.35 mm (red arrow). (**B**) The same data set depicted as box-and-whisker plots showing a significant difference between two groups (A: mean rib width 0.416 mm ± 0.078 SD, and B: 0.289 mm ± 0.037 SD; Mann-Whitney U-test: p ≤ 0.0001), which according to Curry & Endo^16^ should represent the two Atlantic species *T. retusa* (group A) and *T. septentrionalis* (group B). (**C)** Rib widths of *T. retusa* (0.345 mm ± 0.05 SD, n = 15) and *T. septentrionalis* (0,282 mm ± 0.038 SD, n = 10) are still significantly different (Mann-Whitney U-test: p = 0.003), when species are identified based on mitochondrial sequence data (12 S and 16 S rRNA), but the difference is less obvious than in (**B** **)**. Same as in (**C**), but species identification based on nuclear sequence data (28 S rRNA) leading to non-significant differences (Mann-Whitney U-test: p = 0.154) between rib-widths of *T. retusa* (0.335 mm ± 0.049 SD, n = 12) and *T. septentrionalis* (0.298 mm ± 0.059 SD, n = 12).

As in the study of Curry & Endo^16^, length, width and dorso-ventral height of the *Terebratulina* shells as well as ratios between the three size measurements did not discriminate the species because these data formed a gradient from small to large specimens simply reflecting changing size with age (not shown). However, the average rib width over a defined transect (see below and^16^) showed a discontinuity in the resulting plot (Fig. 1A) at about 0.35 mm seemingly reflecting the species boundary between *T. septentrionalis* and *T. retusa*. When displayed as a box-and-whisker plot (Fig. 1B) it was even more obvious that the 40 included specimens (27 IceAGE, 13 MfN brachiopod collection) fell into two significantly different groups, thereby corroborating previous results. The rib width mean value of 0.298 mm representing the group with narrower ribs (=the putative *T. septentrionalis* specimens) was almost identical with the mean value given in Curry & Endo’s study for this species. However, the rib width mean value of the group with broader ribs (=the putative *T. retusa* specimens) was conspicuously smaller than that given by these authors. Nevertheless, the rib width of all our putative *T. retusa* specimens identified by morphology only fell into the variability range of the specimens assigned to *T. retusa* by Curry & Endo, i.e. the distribution of our specimens within this variability range was slightly shifted towards smaller rib widths.

### Molecular analysis

All *Terebratulina* specimens collected during the IceAGE expedition yielded suitable amounts of DNA for sequence analysis. The analysed mitochondrial markers (n = 26) resulted in 6.07% (12 S) and 8.10% (16 S) sequence divergence between two groups of specimens clearly defining them as separate taxa. As we were interested in testing the IceAGE specimens for signs of hybridization, we needed an additional nuclear marker (28 S rRNA) in which potential recombinations may have happened in the past leading to conserved discordancies between the mitochondrial and nuclear genotypes. Thus, we analyzed a 1020 bp long fragment of this marker (n = 25) showing a much lower, but still measurable sequence divergence of 2.25% between the same two groups of specimens. These results confirm that *T. septentrionalis* and *T. retusa* both have species-specific nucleotide signatures, which are intra-specifically conservative and allow for species discrimination on the basis of molecular markers alone.

The critical question now was whether all molecular identifications agree with the clear morphological differentiation on the basis of rib width measurements or whether single specimens showed a combination of sequence identity with one species and morphological affinity to the other species. A second aspect was to look at the combination of both mitochondrial and nuclear markers to check for potential hybrids with discordant species-specific signatures. The sequence alignments of all three markers are given as additional files [see Supplementary alignments S1–S3].

### Morphological and molecular data combined

When combining all results from morphological and molecular analyses (Table 1) it became clear that four different combinations of characters exist among the 25 *Terebratulina* specimens from Iceland successfully sequenced for all three markers in this study. The majority of specimens clearly belonged to either *T. septentrionalis* (first cluster) or *T. retusa* (second cluster) being consistent in all molecular and morphological characters for one or the other species. These were four *T. retusa* (one from station 1034, three from station 1047) and eleven *T. septentrionalis* (one from station 1034, four from station 1047, and six from station 1213). The third cluster comprised 8 specimens (two from station 1034, six from station 1047) which in both mitochondrial and nuclear markers were clearly *T. retusa*, but which showed very narrow shell ribs shifting them into the *T. septentrionalis* morphospace. These specimens, on the basis of morphological characters alone, would certainly have been misidentified in a morphometrical analysis. The fourth cluster is the most interesting one, represented by only two specimens. The rib width of these specimens would clearly identify them as *T. retusa*, as do the mitochondrial markers. But the nuclear marker clearly has the *T. septentrionalis* signature. This mixture of the otherwise highly conserved and species-specific molecular sequences in both mitochondrial and nuclear markers can only be explained by interspecific hybridization. Both specimens with this character mix were homozygous for *T. septentrionalis* in their 28 S rRNA, i.e. they almost certainly do not represent the F1 of a recent hybridization event, but are the result of backcrossings of a hybrid with a parental species, in this case with *T. septentrionalis*. As mitochondrial genes are inherited from the female parent we can even conclude that the original hybridization event happened when a *T. retusa* egg was fertilized by a *T. septentrionalis* sperm. The mitochondrial *T. retusa* signature was then passed on to following generations through the female line.

**Table 1:** Species identification of all IceAGE (Me85/3) samples of *Terebratulina* based on mitochondrial (12 S and 16 S rRNA) and nuclear (28 S rRNA) markers in comparison to species determination based on morphometrical data.

Specimen	12 S/16 S rRNA	28 S rRNA	Morphometry	Accession no.	
1034A	*T. retusa*	*T. retusa*	*T. septentrionalis*	ZMB Bra 2415	3
1034B	*T. retusa*	*T. retusa*	*T. retusa*	ZMB Bra 2416	1
1034C	*T. retusa*	*T. retusa*	*T. septentrionalis*	ZMB Bra 2417	3
1034D	*T. septentrionalis*	*T. septentrionalis*	*T. septentrionalis*	ZMB Bra 2432	2
1047A	*T. retusa*	*T. retusa*	*T. retusa*	ZMB Bra 2418	1
1047B	*T. retusa*	*T. retusa*	*T. retusa*	ZMB Bra 2419	1
1047C	*T. retusa*	*T. retusa*	*T. septentrionalis*	ZMB Bra 2420	3
1047D	*T. septentrionalis*	*T. septentrionalis*	*T. septentrionalis*	ZMB Bra 2430	2
1047E	*T. retusa*	*T. retusa*	*T. retusa*	ZMB Bra 2421	1
1047F	*T. septentrionalis*	*T. septentrionalis*	*T. septentrionalis*	ZMB Bra 2431	2
1047G	*T. retusa*	*T. septentrionalis*	*T. retusa*	ZMB Bra 2428	4
1047H	*T. retusa*	—	*T. septentrionalis*	ZMB Bra 2427	—
1047I	*T. retusa*	*T. septentrionalis*	*T. retusa*	ZMB Bra 2429	4
1047J	*T. retusa*	*T. retusa*	*T. septentrionalis*	ZMB Bra 2422	3
1047K	*T. retusa*	*T. retusa*	*T. septentrionalis*	ZMB Bra 2423	3
1047L	*T. retusa*	*T. retusa*	*T. septentrionalis*	ZMB Bra 2424	3
1047M	*T. retusa*	*T. retusa*	*T. septentrionalis*	ZMB Bra 2425	3
1047N	*T. retusa*	*T. retusa*	*T. septentrionalis*	ZMB Bra 2426	3
1047O	*T. septentrionalis*	*T. septentrionalis*	*T. septentrionalis*	ZMB Bra 2433	2
1047P	*T. septentrionalis*	*T. septentrionalis*	*T. septentrionalis*	ZMB Bra 2434	2
1213A	*T. septentrionalis*	*T. septentrionalis*	*T. septentrionalis*	ZMB Bra 2435	2
1213B	*T. septentrionalis*	*T. septentrionalis*	*T. septentrionalis*	ZMB Bra 2436	2
1213C	*T. septentrionalis*	*T. septentrionalis*	*T. septentrionalis*	ZMB Bra 2437	2
1213D	*T. septentrionalis*	*T. septentrionalis*	*T. septentrionalis*	ZMB Bra 2438	2
1213E	*T. septentrionalis*	*T. septentrionalis*	*T. septentrionalis*	ZMB Bra 2439	2
1213F	*T. septentrionalis*	*T. septentrionalis*	*T. septentrionalis*	ZMB Bra 2440	2

Results of both mitochondrial markers were always alike. Note that specimens 1047G and 1047I have different species-specific molecular signatures in their mitochondrial *versus* nuclear DNA identifying them as hybrids. 1 = *T. retusa*, 2 = *T. septentrionalis*, 3 = *T. retusa* with *septentrionalis*-like shell sculpture, 4 = *T. retusa* x *T. septentrionalis* hybrids.

Consequently, molecular data enable us to divide the samples into bona fide *T. septentrionalis* versus *T. retusa*, which partly contrasts with the previous grouping of specimens “with narrow shell ribs” versus those “with broad shell ribs”. Once the species are identified on the basis of their molecular sequence markers, the resulting box-and-whisker plots of rib widths show that the clear difference between the two morphologically defined clusters becomes much smaller and when using the nuclear sequences for species identification this significant morphological difference even collapses (Fig. 1C,D). This means that the difference between narrow-ribbed and broad-ribbed *Terebratulina* specimens from Iceland is arbitrary and does not reflect a significant difference between *T. septentrionalis* and *T. retusa*. *T. retusa* around Iceland seems to be more variable in this shell character than elsewhere, i.e. some individuals have narrower ribs than the typical *T. retusa* from North Atlantic coasts of mainland Europe.

## Discussion

### Species identification and the hybridization event

As has been shown in a previous study^7^, the molecular markers used here are highly conserved intraspecifically, i.e. identification of either *T. retusa* or *T. septentrionalis* is possible based on sequence information alone. Even seemingly small genetic differences (e.g. 2.25% as in the 28 S rRNA fragment) are sufficient to tell the species apart. The occurrence of sequence information of both species in one individual can be explained if a mixture of these otherwise highly conserved sequences happened in the past. In nature this can be achieved either vertically through hybridization or horizontally through lateral gene transfer. Analyses of full mitochondrial genomes of brachiopods showed no signs of lateral gene transfer^22–25^, so that our observations can only be explained by hybridization between the two species.

### The role of temperature preferences and taxon sampling

The failure to discover the hybrid zone of *T. retusa* and *T. septentrionalis* on the southwestern shelf off Iceland in the otherwise meticulous and well-structured study of Curry & Endo^16^ is simply due to bad luck as their impressive number of specimens analysed supposedly did not contain enough specimens from this critical region. However, their Iceland specimens coded as “b” in their principal component analysis (Fig. 1 in ref. 16) were all identified as *T. retusa* and cluster conspicuously close to the border between the two clouds identified in their data set. This may be interpreted as a hint that *T. retusa* in Iceland has no “typical” morphology, something we could clearly show for at least some of the specimens collected at Meteor stations #1034 and #1047 in the southwest of Iceland. But why is this region so critical? When looking at the temperature regimes around Iceland it is obvious that the southwestern region is influenced by the warm North Atlantic Current leading to temperatures of 7–10 °C throughout the year. In contrast, the water in the northeast of Iceland is much colder due to the East Icelandic Current carrying polar waters southward, leading to annual temperatures of about 5–7 °C (temperature measurements for both regions at 50 m depth by the Icelandic Marine Research Institute in 2011^26–28^). According to Curry & Endo^16^ *T. retusa* prefers temperate water conditions (see also ref. 29), whereas *T. septentrionalis* prefers colder climate (but see ref. 30), the latter being in line with our results that station #1213 revealed only *T. septentrionalis* specimens. Potential hybridization can only occur at water temperatures, which are suitable for both species and this seems to exclude the northeastern region off Iceland. According to the reconstruction of water currents around the island^31^ current mediated transport of *T. septentrionalis* sperm along the clockwise running Iceland coastal current towards the southwestern region may be possible, but seems unlikely due to generally low sperm survival rates in open waters^32^ and the low salinity and variable flow velocity of this coastal current^27^. Rather, our results show that adults of *T. septentrionalis* were found at Meteor stations #1034 and #1047 in the southwestern region, albeit in low frequency, offering the opportunity of crossbreeding through sympatric distribution of the two species.

### The potential influence of life history traits on hybridization

Apart from their specific temperature preferences (see above), *T. retusa* and *T. septentrionalis* also differ in their reproductive biology. *T. septentrionalis* is a brooder, retaining its embryos within the mantle cavity until they have reached an advanced stage of development^33, 34^ (see also ref. 35 for the related Pacific species *T. unguicula*). In contrast, *T. retusa* is a free spawner with both sexes shedding their gametes into the surrounding water. Thus, for a *T. retusa* egg the probability is rather high to be hit by a *T. septentrionalis* sperm, especially if in sympatric populations in Europe the temperature dependent reproductive season of both species is isochronic. On the other hand, larval brooding in the mantle cavity as in *T. septentrionalis* is only possible if (i) sperm is washed into this cavity by the adult’s inhalant feeding current and (ii) spawned eggs ready to be inseminated are retained in the same place. As has been shown in several studies, the inhalant current enters the mantle cavity from left and right sides of the articulate brachiopod shell^36–38^, leading the water through the network of tentacles of both lophophoral arms to filter planktonic particles. Only the filtered and clean water passes through the tentacle network into the mantle cavity and leaves the animal as the exhalant current at the mediofrontal margin of the shell. The brooding female of *T. septentrionalis* must have a sorting mechanism to differentiate between sperm and food as the sperm has to pass the lophophoral tentacles to enter the mantle cavity for inseminating the ripe eggs. This sorting may be accomplished by size selection of the captured particles, as the maximum efficiency for particle capture in *T. retusa* applies to food particles sized 7-8 µm^39^, whereas sperm diameter in *Terebratulina* does not exceed 1.5 µm^40^. If in addition this sorting mechanism is able to differentiate between conspecific and other gametes, *T. retusa* sperm randomly entering the inhalant current of a *T. septentrionalis* female may be doomed. This could be an explanation for finding only descendents of a hybridization event between a *T. retusa* egg and a *T. septentrionalis* sperm in our data. One-way hydridization as assumed here on the basis of different life history traits may be characteristic for this species pair. However, as we have found only two hybrids among our samples, this prediction needs future verification.

### Does the fossil record help?

The oldest fossil brachiopods attributed to the genus *Terebratulina* date back to the Late Jurassic of Europe, California, and New Zealand^41, 42^ basically representing terebratulids with shell ornament. Fossil specimens similar to or identified as *T. retusa* have been reported only from Upper Oligocene to Pleistocene strata in France, Italy, Hungary, Rhodes, and Algeria^16, 43–45^. As described above, extant specimens of *T. retusa* and *T. septentrionalis* are difficult to tell apart on the basis of shell morphology alone, i.e. distinguishing these species as fossils may be even more problematic. This might be the reason for *T. septentrionalis* being absent altogether from the records in the Paleobiology Database^46^. With regard to the unusually high longevity of 60 MY of the two species according to a previous molecular clock approach^7^, the fossil record is, therefore, not suitable to reconstruct the speciation history and palaeogeographic distribution of *Terebratulina* species in the North Atlantic.

## Conclusions

Our results provide the first evidence of hybridization in brachiopods, i.e. between the species *T. retusa* and *T. septentrionalis*. We demonstrated that *T. retusa* in Iceland is much more variable in its shell ornament than previously thought, blurring the clear morphological disparity between the two species assumed by Curry & Endo^16^. The intermediate specimens of Wesenberg-Lund^13–15^ although triggering the search for true hybrids do not necessarily represent descendants of a hybridization event between the two species of *Terebratulina*, but may just reflect the morphological variability of *T. retusa* in Iceland. The scarcity of fossil representatives of the two species and the demonstrated difficulties to identify *T. retusa* and *T. septentrionalis* beyond doubt on the basis of morphometrical characters alone hampers the reconstruction of the speciation event leading to the extant *Terebratulina* species in the North Atlantic. Whereas the genus *Terebratulina* based on the oldest known fossils is of Tethyan origin, its modern representatives in the North Atlantic almost certainly came into being through a vicariance event caused by the opening of the Atlantic Ocean. However, the origin of the Norwegian Finnmarken specimens described as *T. septentrionalis* (see Fig. 3 in ref. 16) remains elusive until molecular data for this isolated population are available.

## Methods

The investigated 28 specimens of *Terebratulina* from Iceland were collected during the IceAGE expedition (RV METEOR Me85/3) in September 2011 at three Stations (#1034, #1047, and #1213) from depths of 209–320 m (Supplementary Fig. S1, Supplementary Table S1). They were collected with Agassiz trawls, picked and preserved in 96% ethanol upon arrival on deck of the vessel. From all samples collected during the cruise, the brachiopods were separated at DZMB Wilhelmshaven and sent to us for further investigation.

### Morphological examination

To maximize comparability with the study of Curry & Endo^16^ we adopted their morphometrical methods and determined shell length, width, and dorso-ventral height with a digital caliper. After dissection of soft tissue for molecular analysis (see below) the 28 Iceland specimens were immersed in 6% sodium hypochlorite and subsequently rinsed in distilled water to get rid of organic tissues and epibionts potentially masking the shell ribs. For photography, shells were mounted dorsal side up in a bed of glass beads and imaged under a Leica Z16 APO Zoom Microscope. Z-Stacks of 12 to 19 photos of the dorsal valve were combined in Auto Montage Essentials v. 5.03 to produce sharp composite images of the dorsal valves. These composites were used for measurements of the rib width in ImageJ, version 1.47. In accordance with Curry & Endo^16^ rib width was measured over a 4 mm transect, lying 4 mm anterior of the dorsal umbo. Ribs were also counted along this transect (Supplementary Fig. S2). For each shell, number of ribs and rib width within the 4 mm transect were measured ten times and mean/standard deviation was calculated. To enlarge the morphometrical dataset we additionally measured 16 *Terebratulina* specimens from the brachiopod dry collection of the Museum für Naturkunde (acronym: ZMB) covering the biogeographical distribution of both, *T. retusa* and *T. septentrionalis* (Supplementary Table S2).

### Molecular analysis

Tissue (lophophore, gonad or musculature) of all 28 Iceland specimens was dried and dissolved in a CTAB mastermix (0.5% 2-mercaptoethanol and 3% proteinase K in CTAB buffer) to extract the mitochondrial and nuclear DNA. Proteins were precipitated with chloroform/isoamyl alcohol, and nucleic acids were precipitated in EtOH with sodium acetate, dried and re-dissolved in 0.1x TE buffer. Mitochondrial sequences were amplified with PCR (GenAmp® PCR system 2700) using primers 12SF1091, 12SR1478, 16SF2510 and 16SR3080^47, 48^, which after sequencing and editing yielded 357 bp fragments of the 12 S rRNA and 429 bp fragments of the 16 S rRNA (n = 26, two samples did not amplify), respectively. All mitochondrial sequences were aligned against the full mt-genome of *T. retusa* (Genbank acc. no. NC_000941.1)^22^. Additionally, nuclear sequences were obtained with specific primers 28SF680, 28SF700, 28SF1062, 28SR1460 and 28SR1797 (see refs 49 and 50 and B.L. Cohen pers. comm.) resulting in an edited alignment of 920 bp (n = 25, with three samples not amplifying) against the 28 S sequence of *T. retusa* published in ref. 50. PCR products were purified using a Nucleospin Kit (Macherey-Nagel, Düren) and commercially sequenced at Services in Molecular Biology GmbH, Rüdersdorf. Sequencing results were edited with BioEdit sequence alignment editor v7.0.0. Sequence alignments of both mitochondrial and nuclear markers were compared between specimens based on single bp comparison across their entire length. This method yielded robust data for species identification even when comparing *Terebratulina* populations across large spatial scales^7^. For PCR primers used see Supplementary Table S3. All sequences obtained were submitted to NCBI and can be identified by their respective Genbank accession numbers according to Supplementary Table S4.

### Statistics

To test for significant differences between morphometric measurements (length, width, thickness and rib width) we used the nonparametric Mann-Whitney U-test (threshold: 5% with p ≤ 0.05) which is appropriate to compare differences between two independent groups when the dependent variable is not normally distributed. Calculations were done with XLSTAT, ver. 2013.5.05 (Addinsoft 1995–2013).

## Electronic supplementary material

Supplementary information
