# Supplementary material for: Detecting hybridization between sister species of Terebratulina (Brachiopoda, Cancellothyridoidea) in the North Atlantic: morphology versus molecules
Source: Sci Rep. 2017 Aug 18;7:8845. doi: 10.1038/s41598-017-09195-0 (PMC5562858; doi:10.1038/s41598-017-09195-0)
Supplement: Supplementary file 1 — Supplementary information [file 41598_2017_9195_MOESM1_ESM.pdf]

Detecting hybridization between sister species of *Terebratulina* (Brachiopoda, Cancellothyridoidea) in the North Atlantic: morphology *versus* molecules

Carsten Lüter*, Nina A. Ebeling, Martin Aberhan

Museum für Naturkunde, Leibniz Institute for Evolution and Biodiversity Science, Invalidenstraße 43, 10115 Berlin, Germany

* corresponding author

Supplementary information

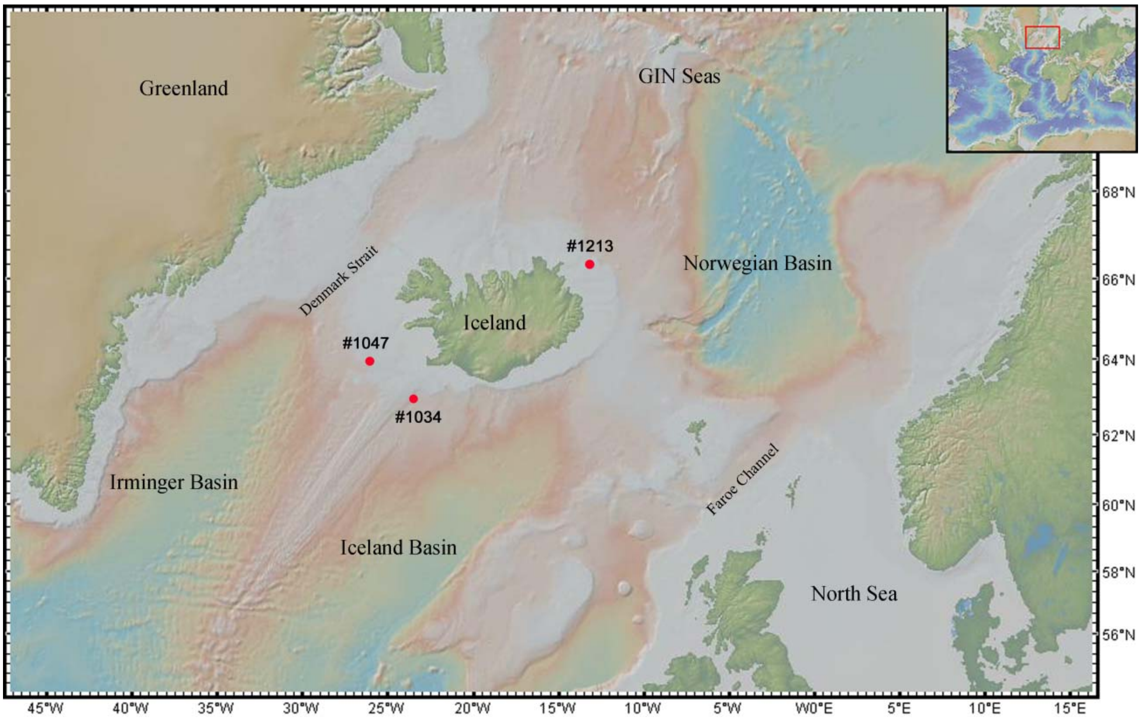

Supplementary Figure S1. Map of the sampling area of the IceAGE expedition (Me85/3) with RV METEOR in September 2011. Red dots represent the three stations where specimens of *Terebratulina* were collected. For details of the Agassiz-trawl stations see Supplementary Table 1. (Map redrawn from [S1], courtesy of Saskia Brix-Elsig).

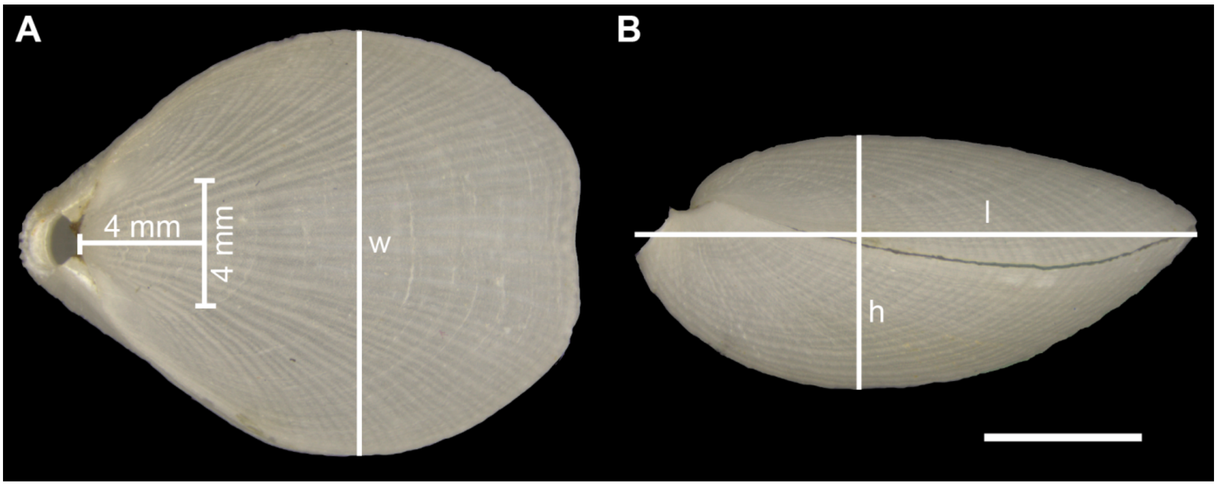

Supplementary Figure S2. Dorsal (A) and lateral (B) views of a shell of *Terebratulina retusa* (ZMB Bra 2421) showing dimensions and positions of measurements used for the morphometric analysis (see text for details). All measurements were taken according to the descriptions in Curry & Endo (see Figs. 1, 2 in [16]) to enable direct comparison with their previous data set. w, shell width; h, shell height; l, shell length; scale bar: 5 mm.

Supplementary Table S1. Coordinates of the three stations at which specimens of *Terebratulina* were collected during the cruise IceAGE (Me85/3) in September 2011.

Station	#1034	#1047	#1213
Location	S Iceland, Reykjanes Ridge, shelf	S Iceland, Irminger Basin, shelf	NE Iceland, Norwegian Sea
Date	4 Sep 2011	6 Sep 2011	22 Sep 2011
Depth (m)	297.2	209.4	318.2
Coordinates	63°19.48'N, 23°9.83'W	63°56.07'N, 25°56.53'W	66°33.16'N, 12°53.40'W

Supplementary Table S2. Provenance of additional specimens from the brachiopod dry collection of Museum für Naturkunde, Berlin used for morphometrical analyses.

Accession no.	No. of specimens	Locality	Ocean
ZMB Bra 334	1	Maine, USA	NW Atlantic
ZMB Bra 1305	1	Casco Bay, USA	NW Atlantic
ZMB Bra 1440	4	Nova Scotia, Canada	NW Atlantic
ZMB Bra 1433	1	Labrador, Canada	NW Atlantic
ZMB Bra 1454	1	Greenland, Denmark	N Atlantic
ZMB Bra 1432	1	Finnmarken, Norway	NE Atlantic
ZMB Bra 237	3	Finnmarken, Norway	NE Atlantic
ZMB Bra 238	3	Finnmarken, Norway	NE Atlantic
ZMB Bra 1338	1	Naples, Italy	Mediterranean

Supplementary Table S3. Markers and their respective primer sequences used in PCR reactions.

Gene	Primer	Primer sequence	Reference
12S	F 1091	5’- AAA AAG CTT CAA ACT GGG ATT AGA TAC CCC ACT AT -3’	[47]
	R 1478	5’- TGA CTG CAG AGG GTG ACG GGC GGT GTG T -3’	[47]
16S	F 16Sar	5’- CGC CTG TTT AAC AAA AAC AT -3’	[48]
	R 16Sbr	5’- CCG GTC TGA ACT CAG ATC ACG T -3’	[48]
28S	F 680	5’- ACC CGC TGA AYT TAA GCA TAT CA -3’	BL Cohen pers. comm.
	F 700	5’- GCA TAT CAA TAA GCG GAG GAA AAG AAA C -3’	[49, 50]
	F 1062	5’- CGT GAA ACC GYT YAG AGG CAA ACG -3’	BL Cohen pers. comm.
	R 1460	5’- AGA CTC CTT GGT CCG TGT TTC AAG AC -3’	BL Cohen pers. comm.
	R 1797	5’- GCT ATC CTG AGG GAA ACT TCG G -3’	[50]

Supplementary Table S4: List of all specimens subject to molecular analyses with ZMB catalogue number, species identification (based on sequence information), collecting station, and Genbank accession numbers. Note that the hybrids (ZMB Bra 2428 and 2429) are labeled as crossbreeds between the two species *T. retusa* and *T. septentrionalis.*

			**Genbank accession numbers**		
**ZMB cat. no.**	**Species**	**Station #**	**12S rRNA**	**16S rRNA**	**28S rRNA**
ZMB Bra 2415	*T. retusa*	1034	MF595951	MF619899	MF619926
ZMB Bra 2416	*T. retusa*	1034	MF595952	MF619900	MF619927
ZMB Bra 2417	*T. retusa*	1034	MF595953	MF619901	MF619928
ZMB Bra 2418	*T. retusa*	1047	MF595954	MF619902	MF619929
ZMB Bra 2419	*T. retusa*	1047	MF595955	MF619903	MF619930
ZMB Bra 2420	*T. retusa*	1047	MF595956	MF619904	MF619931
ZMB Bra 2421	*T. retusa*	1047	MF595957	MF619905	MF619932
ZMB Bra 2422	*T. retusa*	1047	MF595961	MF619909	MF619933
ZMB Bra 2423	*T. retusa*	1047	MF595962	MF619910	MF619934
ZMB Bra 2424	*T. retusa*	1047	MF595963	MF619911	MF619935
ZMB Bra 2425	*T. retusa*	1047	MF595964	MF619912	MF619936
ZMB Bra 2426	*T. retusa*	1047	MF595965	MF619913	MF619937
ZMB Bra 2427	*T. retusa*	1047	MF595959	MF619907	-
ZMB Bra 2428	*T. retusa* x *T. sept.*	1047	MF595958	MF619906	MF619941
ZMB Bra 2429	*T. retusa* x *T. sept.*	1047	MF595960	MF619908	MF619942
ZMB Bra 2430	*T. septentrionalis*	1047	MF595967	MF619915	MF619939
ZMB Bra 2431	*T. septentrionalis*	1047	MF595968	MF619916	MF619940
ZMB Bra 2432	*T. septentrionalis*	1034	MF595966	MF619914	MF619938
ZMB Bra 2433	*T. septentrionalis*	1047	MF595969	MF619917	MF619943
ZMB Bra 2434	*T. septentrionalis*	1047	MF595970	MF619918	MF619944
ZMB Bra 2435	*T. septentrionalis*	1213	MF595971	MF619919	MF619945
ZMB Bra 2436	*T. septentrionalis*	1213	MF595972	MF619920	MF619946
ZMB Bra 2437	*T. septentrionalis*	1213	MF595973	MF619921	MF619947
ZMB Bra 2438	*T. septentrionalis*	1213	MF595974	MF619922	MF619948
ZMB Bra 2439	*T. septentrionalis*	1213	MF595975	MF619923	MF619949
ZMB Bra 2440	*T. septentrionalis*	1213	MF595976	MF619924	MF619950

Supplementary References

S1 Brix, S. et al. The IceAGE project – a follow up of BIOICE. *Pol. Polar Res.* **35**, 141-150 (2014).
